# A severe case of thrombocytopenia, anasarca, fever, renal insufficiency or reticulin fibrosis, and organomegaly syndrome with myocardial and skeletal muscle calcification despite hypocalcemia: a case report

**DOI:** 10.1186/s13256-020-02588-2

**Published:** 2021-01-06

**Authors:** Shogo Minomo, Yu Fujiwara, Shota Sakashita, Akito Takamura, Kaoru Nagata

**Affiliations:** 1 Department of General Internal Medicine, Musashino Red Cross Hospital, 1-26-1, Kyonancho, Musashino-shi, Tokyo, 1808610 Japan; 2 Department of Medicine, Icahn School of Medicine at Mount Sinai, Mount Sinai Beth Israel, 281 First Avenue, New York, NY 10003 USA; 3 Department of Medical Oncology, Musashino Red Cross Hospital, 1-26-1, Kyonancho, Musashino-shi, Tokyo, 1808610 Japan; 4 Department of Nephrology, Musashino Red Cross Hospital, 1-26-1, Kyonancho, Musashino-shi, Tokyo, 1808610 Japan; 5 Department of Rheumatology and Collagen Disease, Musashino Red Cross Hospital, 1-26-1, Kyonancho, Musashino-shi, Tokyo, 1808610 Japan

**Keywords:** TAFRO syndrome, Myocardial calcification, Skeletal muscle calcification, Hypocalcemia, Tocilizumab

## Abstract

**Background:**

TAFRO (thrombocytopenia, anasarca, fever, renal insufficiency or reticulin fibrosis, and organomegaly) syndrome is a recently recognized disease with a variety of presentations of variable severity. In acute settings, this disease also involves organ dysfunction because of the associated systemic inflammation. However, cases of TAFRO syndrome with myocardial and/or skeletal muscle calcification have never been reported.

**Case presentation:**

A 24-year-old healthy young Asian man was admitted with intermittent epigastric pain and fever for 2 weeks. Computed tomography revealed pleural effusion, ascites and systemic lymphadenopathy. Laboratory tests showed thrombocytopenia, elevated C-reactive protein, hypoalbuminemia, anemia and renal dysfunction. Based on these findings and bone marrow biopsy, we diagnosed his disease as TAFRO syndrome and commenced hemodialysis for the renal dysfunction. However, he developed refractory hypocalcemia with unstable vital signs, for which we administered calcium gluconate hydrate. Thereafter, myocardial and skeletal muscle calcification was revealed radiologically, with the myocardial calcification causing sick sinus syndrome. He was treated with tocilizumab and finally discharged in an ambulatory condition after prolonged hospitalization, with residual calcific lesions.

**Conclusion:**

This is the first report of a patient with TAFRO syndrome and the complication of organ calcification. The etiology of calcification in this case is not clear. Systemic inflammation with possible hypercytokinemia might have been involved in the unexpected complication of systemic calcification. It is important to carefully handle the general management of TAFRO syndrome because of the possibility of various complications.

## Background

TAFRO (thrombocytopenia, anasarca, fever, renal insufficiency or reticulin fibrosis, organomegaly) syndrome is a systemic inflammatory disease first reported by Takai et al. in 2010 [1], and is categorized as a subtype of idiopathic multicentric Castleman’s disease (iMCD). TAFRO syndrome follows a particularly aggressive clinical course compared to unicentric Castleman’s disease or other subtypes of iMCD. Therefore, clinicians often experience difficulty in treating this condition. Since its earliest description, reports of cases of TAFRO syndrome have been described worldwide, and guidelines for its treatment have been proposed [2–5]. Since organ dysfunction is commonly seen with TAFRO syndrome, temporary hemodialysis is often necessary during the course of treatment of this condition [2], although no case reports have described calcific deposits associated with hemodialysis. Additionally, although several cases with severe cardiac dysfunction due to chemotherapy, hypercytokinemia and massive ascites have been reported [6–8], there have been no cases of gross calcification recognized radiologically, either in the myocardium or in skeletal muscles.

We report a case of TAFRO syndrome with myocardial and skeletal muscle calcification, who was finally discharged from the hospital in an ambulatory condition following tocilizumab therapy. Our patient also presented with severe hypocalcemia during his clinical course, the treatment of which might have eventually triggered systemic calcification. Although it is difficult to completely understand the etiological mechanism in this case, our experience will contribute to understanding the pathophysiology and general management of TAFRO syndrome.

## Case presentation

**Fig. 1:**
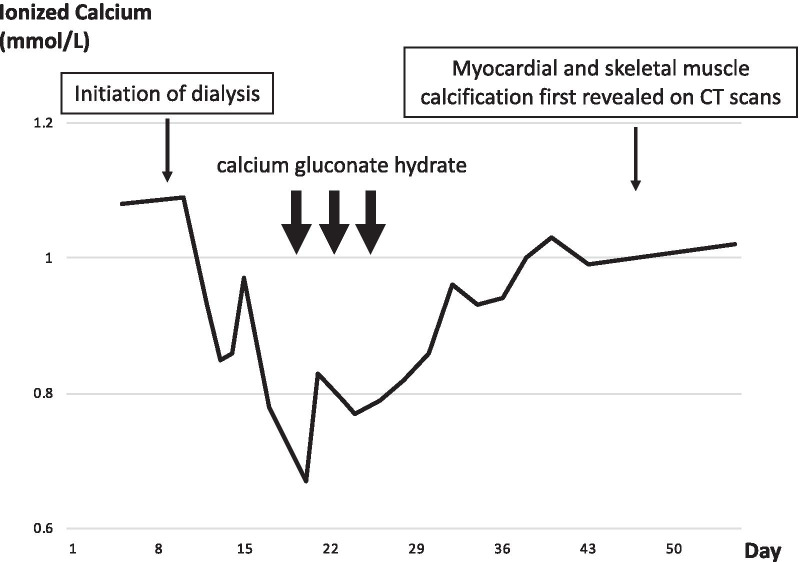
Transition of ionized calcium. Hypocalcemia appeared after the initiation of dialysis, and myocardial/skeletal muscle calcification was revealed on CT scans after the recovery from hypocalcemia with administration of calcium gluconate hydrate

**Fig. 2:**
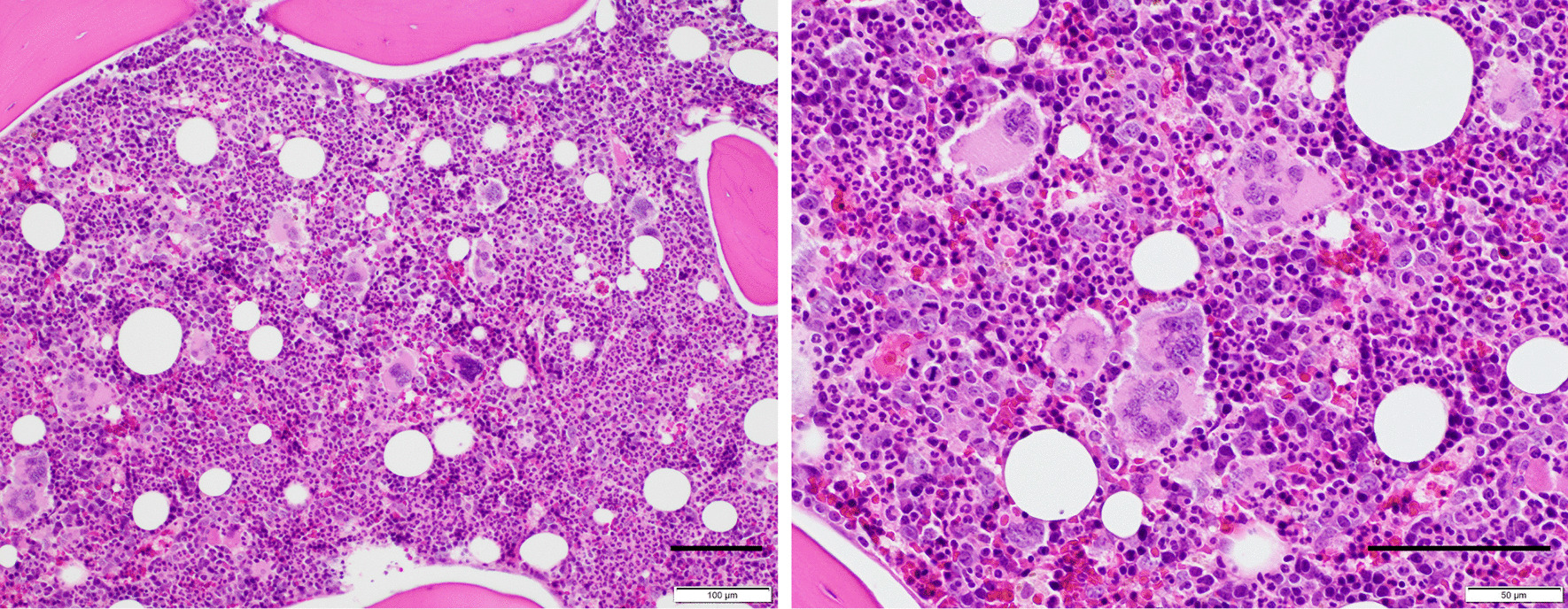
Histological findings on bone marrow biopsy. **a**, **b** Hypercellular marrow with increased megakaryocytes are seen. (Hematoxylin and eosin staining). (Scale bar, 100 μm)

A previously healthy 24-year-old Asian man without significant family medical history presented with intermittent severe epigastric pain and fever for two weeks. Since a computed tomography (CT) scan at another hospital had only revealed systemic lymphadenopathy and could not detect any cause for his symptoms, he was referred to our hospital for further investigation. On admission, he was febrile (37.4 °C). Physical examination showed epigastric tenderness without signs of peritoneal irritation, and pitting edema on the lower extremities. His laboratory test results on admission are shown in Table 1. Contrast-enhanced CT scan revealed cervical, supraclavicular, axillary, paraaortic and inguinal lymphadenopathies, bilateral pleural effusion, ascites and hepatosplenomegaly. Soon after his admission, hemodialysis and mechanical ventilation were initiated due to the development of anuria and dyspnea, and methylprednisolone pulse therapy (1 g/day) was administered for three days after ruling out bacterial infections on hospital day 9, followed by intravenous methylprednisolone (50 mg/day) as maintenance therapy. Laboratory data on hospital day 11 are shown in Table 2. In spite of hemodialysis and ventilatory support, his vital signs were unstable, necessitating temporary use of catecholamines to maintain his blood pressure. Additionally, his plasma calcium level decreased (ionized calcium: 0.78 mmol/L) with a high level of phosphorus (9.6 mg/dL) on hospital day 17. Subsequently, we intravenously administered calcium gluconate hydrate for a total of eight days because the hypocalcemia was refractory to therapy. Laboratory data indicated low vitamin D and high intact parathyroid hormone levels, as seen in patients with chronic kidney disease. Finally, his plasma calcium levels were maintained within the normal range (Fig. 1). Bone marrow biopsy revealed hypercellular marrow with an increased number of megakaryocytes (Fig. 2). Based on these findings and exclusion criteria, we diagnosed his condition as TAFRO syndrome according to the diagnostic criteria proposed in 2015 [3].

**Table 1:** Laboratory data on admission

Complete blood cell count			Blood chemistry		
WBC	14,100	/μL	Total protein	5.1	g/dL
Seg	83	%	Albumin	1.8	g/dL
Eosino	1	%	T.Bil	1.0	mg/dL
Lympho	7	%	AST	29	IU/L
Mono	9	%	ALT	14	IU/L
RBC	413	10^4^/μL	LDH	370	IU/L
Hgb	13.5	g/dL	ALP	265	IU/L
MCV	86.2	fL	γ-GTP	105	IU/L
Platelet	46.8	10^4^/μL	BUN	43.1	mg/dL
			Cr	1.62	mg/dL
Coagulation system			eGFR	46	mL/min/1.73m^2^
PT	18.2	sec	CRP	25.37	mg/dL
PT-INR	1.51		Glu	78	mg/dL
APTT (standard: 26-36)	47.9	sec	AMY	39	IU/L
Fibrinogen	>600	mg/dL			
D-dimer	26.9	μg/mL			

*WBC* White blood cell, *RBC* Red blood cell, *Hgb* Hemoglobin, *MCV* Mean corpuscular volume, *PT* Prothrombin time, *PT-INR* Prothrombin time-international normalized ratio, *APTT* Activated partial thromboplastin time, *T.Bil* Total Bilirubin, *AST* Aspartate aminotransferase, *ALT* Alanine aminotransferase, *LDH* Lactate dehydrogenase, *ALP* Alkaline phosphatase, *BUN* Blood urea nitrogen, *Cr* Creatinine, *CRP* C-reactive protein, *AMY* Amylase

**Table 2:** Laboratory data at the time of diagnosis of TAFRO syndrome

Complete blood cell count			Blood chemistry			Immunologic test		
WBC	23,600	/μL	Albumin	1.5	g/dL	IgG	1385	mg/dL
Band	6	%	T.Bil	0.4	mg/dL	IgG4	66	mg/dL
Seg	88	%	AST	33	IU/L	IgA	187	mg/dL
Lympho	3	%	ALT	12	IU/L	IgM	62	mg/dL
Mono	3	%	LDH	471	IU/L	ANA	<40	times
RBC	348	10^4^/μL	γ-GTP	266	IU/L	anti-RNP Ab	–	
Hgb	9.9	g/dL	ALP	400	IU/L	anti-ds-DNA IgG	–	
MCV	86.8	fL	BUN	119.5	mg/dL	anti-SS-A Ab	–	
Platelet	7.5	10^4^/μL	Cr	7.77	mg/dL	RF	<3	IU/mL
ESR	87	mm/hr	CRP	36.35	mg/dL	PR3-ANCA	<1	IU/mL
			Ferritin	1873	ng/mL	MPO-ANCA	<1	IU/mL
			ACE	7.6	IU/L	Coombs test	–	
			Haptoglobin	311	mg/dL	PA-IgG	70	ng/10^7^cells
Infectious diseases						ADAMTS-13	28	%
EBV VCA IgG	+		Coagulation system			anti-GBM Ab	<2	U/mL
EBV VCA IgM	-		PT	19	Sec	sIL-2R	1260	U/mL
EBNA IgG	+		PT-INR	1.59		IL-6	65.1	pg/mL
CMV C7-HRP	-		APTT	>80	Sec	VEGF	1030	pg/mL
			(Standard: 26-36)					
HIV antigen/Ab	-		Fibrinogen	>600	mg/dL			
HHV-8 DNA (WBC10^6^cells)	<20		AT-III	58	%			
IGRA	–		FDP	66	μg/mL			

*WBC* White blood cell, *RBC* Red blood cell, *Hgb* Hemoglobin, *MCV* Mean corpuscular volume, *ESR* Erythrocyte sedimentation rate, *EBV* Epstein–Barr virus, *VCA* Virus capsid antigen, *EBNA* Epstein–Barr virus nuclear antigen, *CMV* Cytomegalovirus, *HIV* Human immunodeficiency virus, *Ab* Antibody, *HHV-8* Human herpesvirus-8, *IGRA* Interferon-gamma releasing assay, *T.Bil* Total Bilirubin, *AST* Aspartate aminotransferase, *ALT* Alanine aminotransferase, *LDH* Lactate dehydrogenase, *γ-GTP* Gamma-glutamyl transpeptidase, *ALP* Alkaline phosphatase, BUN Blood urea nitrogen, *Cr* Creatinine, *CRP* C-reactive protein, *ACE* Angiotensin-converting enzyme, *PT* Prothrombin time, *PT-INR* Prothrombin time-international normalized ratio, *APTT* Activated partial thromboplastin time, *AT-III* Antithrombin III, *FDP* Fibrin/fibrinogen degradation products, *ANA* Antinuclear antibody, *anti-RNP-antibody* Anti-ribonucleoprotein antibody, *anti-ds-DNA IgG* Anti-double-stranded deoxyribonuclein acid immunoglobulin G, *anti-SS-A antibody* Anti-Sjören's syndrome A antibody, *RF* Rheumatoid factor, *PR3-ANCA* Proteinase 3-anti-neutrophil cytoplasmic antibody, *MPO-ANCA* Myeloperoxidase-anti-neutrophil cytoplasmic antibody, *PA-IgG* Platelet-associated immunoglobulin G, *anti-GBM antibody* Anti-glomerular basement membrane antibody, *sIL-2R* Soluble interleukin-2 receptor, *IL-6* Interleukin-6, *VEGF* Vascular endothelial growth factor

**Fig. 3:**
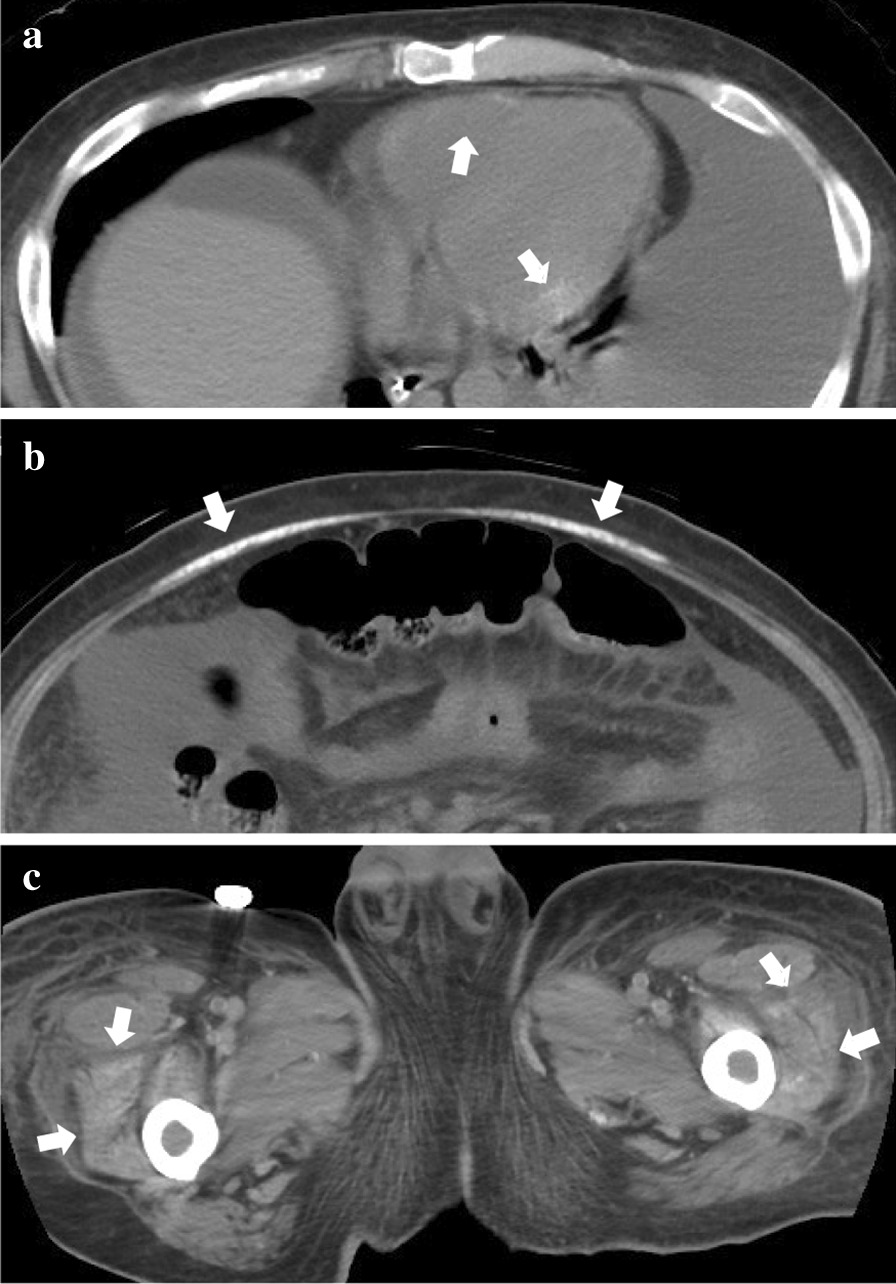
Myocardial and skeletal muscle calcification noted on computed tomography scans for the first time. **a** High density areas were present on the myocardium (arrows). **b**, **c** The rectus abdominis muscle and those of the thigh (arrows) showed high density bilaterally

**Fig. 4:**
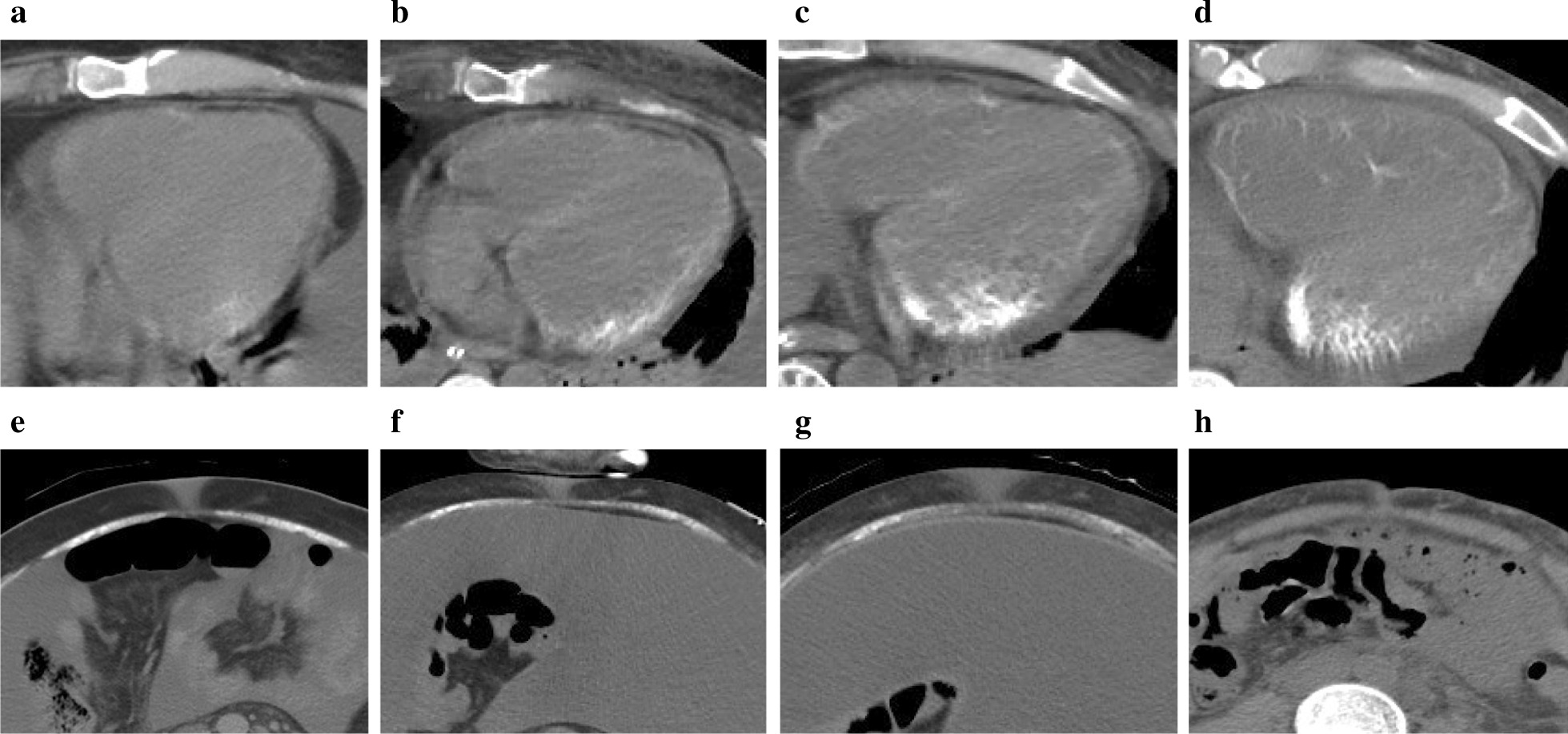
Chronological changes in myocardial and skeletal muscle (rectus abdominis) calcification on computed tomography images. Computed tomography was performed on (**a**, **e**) day 47, (**b**, **f**) day 123, (**c**, **g**) day 177 and (**d**, **h**) day 345. The degree of calcification showed progressive worsening during the first few months; subsequently, myocardial calcification remained unchanged, while that in the skeletal muscle gradually diminished

We initiated weekly treatment with the biologic tocilizumab (8 mg/kg), which is a recombinant, humanized, anti-human interleukin-6 (IL-6) receptor monoclonal antibody, along with tapering glucocorticoids on hospital day 32. However, on hospital day 76, he presented with syncope due to sick sinus syndrome. We first recognized myocardial and skeletal muscle calcification by CT scans on hospital day 47 (Fig. 3). Subsequent CTs showed progressive worsening of the degree of calcification in the first few months (Fig. 4). At this time, we could not perform temporary pacing because of the risk of hemorrhage due to persistently low platelet levels and coagulopathy.

Although he repeatedly suffered from various infections (such as catheter related blood stream infection, pyothorax, intraabdominal abscess and infectious endocarditis), we basically continued use of tocilizumab, except when the infection was very severe. We initiated romiplostim, an analog of thrombopoietin, on day 173 because his platelet count remained persistently low, which soon resulted in recovery of platelet count to within the normal range. In addition, urine volume began to gradually increase five months after the onset of anuria. Thereafter, calcification of the rectus abdominis muscle also began to gradually diminish, as observed by CT scans, although there were no remarkable changes in that of the myocardium (Fig. 4).

**Fig. 5:**
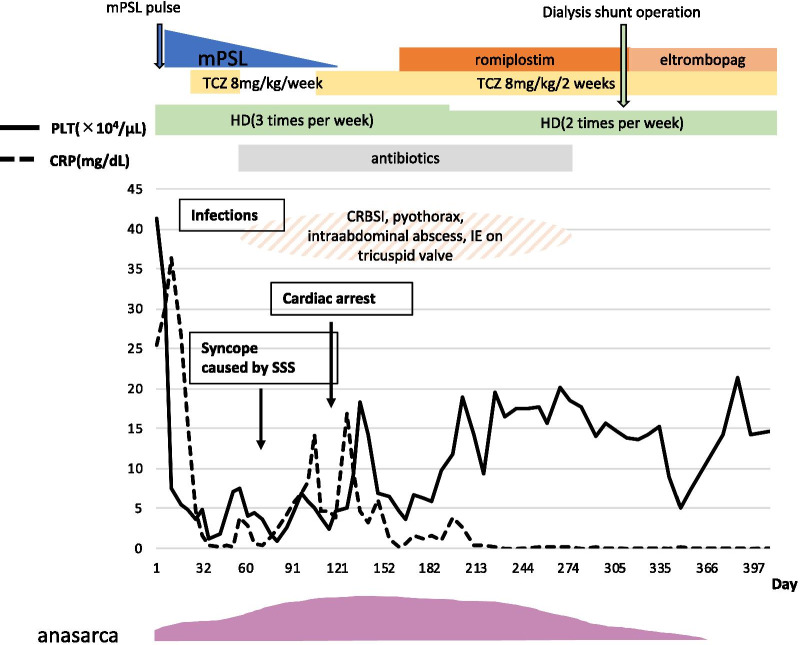
Clinical course from admission until discharge. *PLT* platelets, *CRP* C-reactive protein, *mPSL* methylprednisolone, *TCZ* tocilizumab, *HD* hemodialysis, *CRBSI* catheter-related blood stream infection, *IE* infectious endocarditis, *SSS* sick sinus syndrome

Although there were many life-threatening events throughout his prolonged hospitalization, he was finally discharged from the hospital in an ambulatory condition on hospital day 410 (his complete clinical course is shown in Fig. 5). Sick sinus syndrome resolved together with improvement in his general condition, so permanent pacemaker implantation was deemed unnecessary. Currently, he receives regular hemodialysis and intravenous tocilizumab every three weeks without evidence of recurrence for one year after discharge.

## Discussion

### TAFRO syndrome and cardiac events

TAFRO syndrome is defined as a subtype of idiopathic multicentric Castleman’s disease (iMCD) that has been recognized as having a severe clinical course. Masaki et al. proposed the diagnostic criteria and severity classification in 2015, and there have been several recently reported clinicopathological analyses and consensus guidelines for the treatment of iMCD based on the accumulated data [2–5]. Our case fulfilled the diagnostic criteria of TAFRO syndrome according to the guideline, and was classified as grade 4 (severe) severity. Our case is rare not only because of many life-threating events such as repetitive severe infections, sick sinus syndrome and cardiac arrest, but also because of the myocardial and skeletal muscle calcification that occurred two months after his disease onset. Other cases have also survived despite a very severe clinical course [9–11], although none of them experienced systemic calcification.

Many TAFRO syndrome cases have recovered with the use of biological products (such as tocilizumab or rituximab), glucocorticoids, immunosuppressants (such as cyclosporine A or sirolimus), and occasionally, with chemotherapy. In terms of cardiac dysfunction, Yasuda et al. reported two cases of TAFRO syndrome with cardiomyopathy and lowered ejection fraction (EF) [6]. They performed CHOP (cyclophosphamide, adriamycin, vincristine and prednisolone) therapy as treatment, and assumed that the cardiomyopathy was an adverse effect of adriamycin. Hiramatsu et al. showed a case of TAFRO syndrome with reversible cardiomyopathy, in which they opined that the high concentration of plasma IL-6 was the cause of cardiomyopathy [7]. However, these cases did not have any associated calcification.

### The etiology of myocardial/systemic calcification

Myocardial calcification itself only occurs very rarely. There are traditionally two pathways causing myocardial calcification: metastatic and dystrophic. Metastatic calcification occurs with hypercalcemia and/or abnormality of calcium/phosphate metabolism [12]. This form of calcification can occur anywhere in the body, but is more likely in areas of high alkalinity, such as the gastric mucosa or systemic arteries; therefore, calcification due to this etiology can exist systemically. On the other hand, dystrophic calcification occurs secondary to cellular damage and necrosis, with calcium deposits replacing necrotizing cells. Other etiologies of dystrophic calcification include trauma, infections, inflammation, neoplasms and drugs [12]. Myocardial infarction is a common cause of myocardial calcification. Septic shock is also a cause of this form of calcification; hypotension during shock and catecholamine use can cause myocardial damage, which eventually results in calcium deposits even if plasma calcium concentration is normal [12, 13]. In our case, the etiology of myocardial and skeletal muscle calcification was considered more likely to be metastatic than dystrophic due to the following reasons. First, there was a failure of calcium/phosphate metabolism due to the presence of progressive renal dysfunction with hemodialysis and low vitamin D levels. There was also excessive replenishment of calcium, with an amount of 7.8 mmol of calcium gluconate hydrate per day on average, which could have accelerated calcium deposition in systemic tissues. We did not administer intravenous vitamin D agents, except for the minimal amount of vitamins administered through total parenteral nutrition, which might have been a possible cause for prolonged low calcium levels. Second, both myocardial and skeletal muscle calcification were detected on CT scans at the same time, which was within a month after the initial identification of hypocalcemia and subsequent overloading of calcium (Fig. 3). Third, although we temporarily used catecholamines, there were no other conditions explaining the dystrophic calcification, such as septic shock or myocardial infarction. Fourth, hypercytokinemia, including elevated IL-6 caused by TAFRO syndrome, could also have contributed to this unusual situation. Plasma calcium levels remained low and were almost unchanged when calcium gluconate hydrate was administered, which suggested that the administered calcium probably leaked out of the vessels and was absorbed by peripheral tissues. Increase of vasopermeability is another possible etiology for the calcified deposits, which could explain its occurrence in the absence of hypercalcemia.

In conclusion, this case of TAFRO syndrome achieved a successful recovery with tocilizumab therapy along with prolonged hospitalization, although he experienced the unexpected complication of myocardial and skeletal muscle calcification probably via a metastatic pathway triggered by drug administration. In acute clinical settings, the tendency is to urgently correct abnormal laboratory data, especially electrolyte abnormalities. However, TAFRO syndrome is known to be associated with hypercytokinemia and organ dysfunction with an unknown pathophysiology; hence, the general management of such cases should be carefully handled, anticipating unexpected complications.

## Data Availability

The datasets used and/or analyzed during the current study are available from the corresponding author on reasonable request.

## Abbreviations

TAFRO: Thrombocytopenia, anasarca, fever, renal insufficiency or reticulin fibrosis, and organomegaly
iMCD: Idiopathic multicentric Castleman’s disease
CT: Computed tomography
IL-6: Interleukin-6
CHOP: Cyclophosphamide, adriamycin, vincristine and prednisolone
